# Bidirectional crosstalk between sleep disorders and gynecological cancers: unraveling molecular synergies and precision therapeutics

**DOI:** 10.3389/fmed.2025.1717587

**Published:** 2025-11-21

**Authors:** Hongxia Mei, Chenyu Zhao, Hongyu Jin, Weiyi Qi, Xiangqun Lu, Yiqing Xin, Wei Wang, Yakai Sun, Wen-Yang Li

**Affiliations:** 1 Department of Respiratory and Critical Care, The First Hospital of China Medical University, Shenyang, China; 2 Department of China Medical University, The Queen’s University of Belfast Joint College, School of Pharmacy, China Medical University, Shenyang, China; 3 School of Pharmacy, Queen’s University Belfast, Belfast, United Kingdom; 4 School of Pharmacy, Jilin University, Changchun, China

**Keywords:** sleep disorders, obstructive sleep apnea, gynecological cancers, circadian rhythm, hypoxia-inducible factor, tumor micro-environment

## Abstract

Sleep disorders, particularly obstructive sleep apnea (OSA), circadian disruption, and insomnia, are increasingly recognized as contributors to the onset and progression of gynecologic cancers. This review explores the bidirectional interactions between sleep dysfunction and malignancies such as ovarian, endometrial, and cervical cancers. Mechanistically, intermittent hypoxia (IH) from OSA promotes tumor aggressiveness through hypoxia-inducible factor-1 alpha (HIF-1α) stabilization, M2 macrophage polarization, and impaired DNA repair, while circadian disruption alters endocrine signaling and immune regulation. Disrupted sleep also perturbs the gut and vaginal microbiota, promoting systemic inflammation and tumor-supportive environments. Conversely, cancer therapies such as chemotherapy and radiotherapy exacerbate sleep dysfunction via neurotoxicity and fibrotic airway damage, especially in estrogen-deprived states. These interconnected mechanisms not only worsen clinical outcomes but also underscore sleep as a modifiable and actionable therapeutic target. Emerging integrative strategies—such as hypoxia-targeted nanomedicine, circadian-based chronotherapy, and microbiota modulation—offer promising avenues to enhance treatment efficacy and quality of life. Progress in this field hinges on interdisciplinary collaboration and the development of personalized care models that embed sleep health as a core component of gynecologic cancer management.

## 1 Introduction

Sleep is fundamental physiological process essential for maintaining systemic homeostasis. It regulates immune function, supports cardiovascular stability, and maintains metabolic balance, while also playing a pivotal role in cognitive performance and emotional regulation (1). Disruptions in sleep architecture—such as insomnia, circadian rhythm sleep–wake disorders, sleep-related movement disorders, sleep disordered breathing—compromise these functions, leading to poorer health outcomes and reduced quality of life, particularly during hormonal dynamic periods such as menstrual cycle, pregnancy, and menopause (2, 3). Obstructive sleep apnea (OSA), characterized by recurrent episodes of partial or complete upper airway collapse during sleep, is a prevalent but historically underdiagnosed condition in women. Importantly, growing epidemiological evidence links OSA in women not only with cardiometabolic and cognitive sequelae but also with an elevated risk for site-specific malignancies, particularly gynecological cancers (4). Despite these associations, research explicitly investigating OSA in gynecologic cancer cohorts remains limited. Most existing studies have focused on breast cancer, which is often included in gynecologic analyses due to overlapping hormonal pathways, such as estrogen receptor signaling (5). This lack of targeted data highlights the need for dedicated studies exploring the prevalence, mechanisms, and clinical implications of sleep disorders in ovarian, cervical, endometrial, and other gynecologic cancer sub-types. Therefore, this review aims to synthesize the existing evidence and propose a conceptual framework specifically focusing on the bidirectional crosstalk between sleep disorders (with an emphasis on OSA) and gynecological cancers, to spur further mechanistic and clinical research in this under-explored area.

### 1.1 Sleep disorders and obstructive sleep apnea in women

OSA, as a representative of sleep disordered breathing, has garnered increasing attention for its dual role in triggering intermittent hypoxia (IH) and contributing to excessive daytime sleepiness and frequent arousals (6, 7). It is noteworthy that the prevalence of OSA varies significantly across age groups and genders. While the overall prevalence in the general population ranges from 9 to 38%, rates are particularly higher among older adults, with some studies reporting rates as high as 78% in elderly women (4), while traditionally considered a male-dominated disorder, significant prevalence exists in females, particularly during peri-menopause and post-menopause.

OSA prevalence in women varies significantly across the lifespan, influenced by hormonal status. During reproductive years, women exhibit roughly a threefold lower prevalence compared to age-matched men (approximating a 1:3 ratio), and this protection is largely attributed to hormonal influences—specifically, progesterone and estrogen—which enhance upper airway stability and reduce ventilatory instability (8–11). In women with concurrent obesity, obstructive sleep apnea (OSA), and endometrial cancer, adipokines (leptin/adiponectin) and estrogen pathways collectively regulate metabolic dysfunction (12–14). ≥50% of their survivor patients were overweight/obeseImportantly, this hormonal benefit is modulated by factors like obesity and endocrine disorders. At menopause, with declining sex hormones, OSA prevalence in women substantially increases, approaching rates seen in men (15). Circadian rhythm disorder can lead to abnormal hormone secretion, which is associated with an elevated risk of fatal ovarian cancer (RR = 1.27). This life-stage variation strongly implicates the critical role of sex hormones in OSA pathogenesis. The prevalence of OSA during pregnancy is 10.5–33.3%, which is closely related to diaphragm elevation caused by uterine enlargement, congestion and edema of airway mucosa caused by estrogen and progesterone, and weight gain (16–18). The prevalence of OSA increases significantly in perimenopausal and postmenopausal women, primarily due to the decline in sex hormone levels (19, 20). On the other hand, OSA patients are at high risk of circadian disruption (21). Such circadian disruption impairs the regulation of endocrine factors governed by the hypothalamic–pituitary axis, including cortisol, growth hormone (GH), prolactin (PRL), thyroid hormones and sex steroids (22). Receptors for female hormones such as estradiol and progesterone are widely distributed in sleep–wake regulatory brain regions, including the basal forebrain, hypothalamus, dorsal raphe nucleus, and locus coeruleus (23). Although the incidence and underlying causes of OSA vary among women of different age groups, these hormonal influences may help explain why sex hormones are consistent risk factors for OSA across the female lifespan. Clinically, investigating the features of OSA in women may elucidate sex-specific health disparities, thereby improving its diagnosis and management.

### 1.2 Sleep disorders, OSA and gynecological cancer

Gynecological cancers including cervical, endometrial, ovarian, and vulvovaginal cancer have exhibited a rising global incidence and mortality over the last three decades (24). In parallel, sleep disorders have emerged as increasingly prevalent comorbidity that may influence both the development and progression of these malignancies. Disruptions in sleep, particularly circadian rhythm disorders, insomnia, and OSA, are recognized for their potential role in tumor biology. Short sleep duration has been implicated in the pathogenesis of hormone-dependent malignancies, including breast, endometrial, and ovarian cancers, with meta-analyses indicating a modest association between insufficient sleep and cancer incidence (HR = 1.06) (25, 26). Nevertheless, these findings warrant cautious interpretation due to ongoing inconsistencies and methodological limitations across observational studies (27). Meta-analysis by Lu et al. (28) (10 studies) found no significant association between sleep duration and overall cancer risk, though a marginal trend linked short sleep to ovarian cancer development. Emerging pathophysiological models suggest that psychological stressors and insomnia may synergistically disrupt cervicovaginal microbial homeostasis. This dysbiotic state may promote chronic inflammation and enhance the oncogenic potential of human papillomavirus (HPV), potentially accelerating the progression from viral persistence to cervical carcinogenesis (21).

In addition to disruptions in sleep, OSA has been increasingly recognized as a potential risk factor influencing cancer initiation, progression and prognosis (29). Clinical data robustly associate OSA with cancers like breast and prostate (28). The clinical relevance of OSA in oncology was first highlighted by the Spanish Sleep Network in 2013, which identified nocturnal hypoxemia-quantified by Tsat90% (the percentage of nighttime with oxygen saturation <90%)-as a significant predictor of increased cancer incidence (30). Subsequent large-scale studies have reinforced these findings. In a cohort of 34,848 OSA patients versus 77,380 controls, OSA conferred a 53% elevated overall cancer risk (RR = 1.53), with site-specific risks including a 2.09-fold increase in breast cancer (HR = 2.09) (31–33). Notably, severe OSA (apnea-hypopnea index, AHI ≥30) correlates with a 4.8-fold higher cancer mortality risk (HR = 4.8), a relationship that strengthens when Tsat90% replaces AHI as the severity marker (HR = 8.6) (34). These risks are further amplified in cancer survivors, where sleep disorders like insomnia contribute to treatment delays and immune dysfunction, perpetuating a vicious cycle of disease progression (35).

Although it is evident that sleep disorders, especially OSA, are closely related to gynecological cancers, research on the underlying biological mechanisms remains relatively limited. Hormones have been shown to play contributory roles in the development and progression of hormone-sensitive cancers, including breast cancer, prostate cancer, endometrial cancer, and ovarian cancer (36, 37). Circadian rhythm disorder can lead to abnormal hormone secretion, which is associated with an elevated risk of fatal ovarian cancer (RR = 1.27) (38). Moreover, women using estrogen therapy have been reported to exhibit a 40% lower incidence of ovarian cancer compared to postmenopausal women not using hormone replacement (39). Additionally, urinary melatonin levels may be associated with the risk of developing ovarian cancer, suggesting a potential link between circadian regulation and tumorigenesis (OR = 0.79) (40). Physiological and pathological fluctuations in key hormones—including progesterone, melatonin, and cortisol—are significant drivers of circadian disruption in women (41–43). This disruption manifests as altered expression of circadian rhythm hormones (e.g., melatonin itself), core clock genes (e.g., Clock, Bmal1, Per, Cry), and downstream genes regulating growth control and reproductive pathways (44). Critically, the resulting dysregulation reciprocally exacerbates hormonal imbalance, indicating a bidirectional relationship between circadian dysfunction and the endocrine system. Consequently, this established endocrine-circadian axis provides a fundamental mechanistic link connecting chronic sleep disorders like OSA to cancer development and progression.

On the contrary, sleep disturbances are highly prevalent among gynecologic oncology patients, with reported prevalence rates ranging from 30 to 88%—substantially higher than the 4 to 33% observed in the general population and the 30 to 50% reported among general cancer survivors (45–52). Regardless of the specific type of gynecologic cancer, many women experience sleep disturbances around the time of diagnosis (50). In postmenopausal women with breast or endometrial cancer, the prevalence of OSA is notably high, with 58 and 57% of patients, respectively, exhibiting and AHI greater than 15 events/hour (5). Proposed mechanisms of the development of sleep disturbances include the release of cytokines such as tumor necrosis factor (TNF) or C-reactive protein (CRP), depression and distress, cancer-related fatigue, menopausal hormone therapy (MHT), chemotherapy, radiotherapy, and surgery (50, 53–55).

An in-depth understanding of gynecological tumors and the discussion on the role and mechanism of tumor and their treatments in sleep and sleep respiratory disorders is conducive to the formulation of clinical comprehensive management pathways.

## 2 Molecular mechanisms of sleep disorder-induced cancer initiation and progression

### 2.1 Circadian disruption and genomic instability

**Figure 1:**
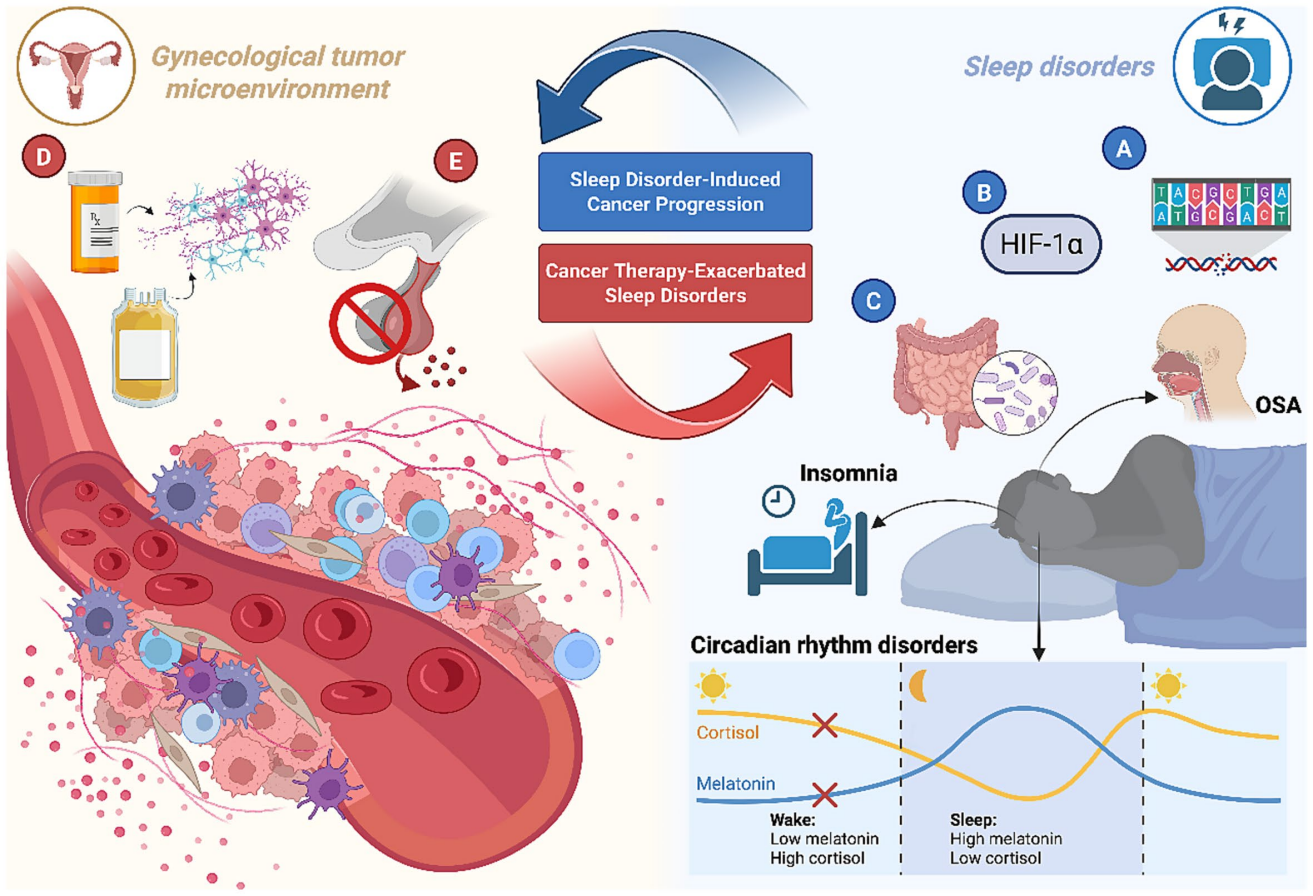
Molecular mechanisms of sleep disorder-induced cancer progression. **(A)** Disruption of circadian rhythms leads to genomic instability. **(B)** OSA-associated IH produces distinct HIF isoform regulation, with preferential stabilization of HIF-1α. **(C)** OSA-related hypoxia induces intestinal hyperpermeability and gut dysbiosis. **(D)** CIPN constitutes structural and functional damage to peripheral nerves. **(E)** Hormonal regimens aim to suppress systemic estrogen via aromatase inhibitors. Created with Biorender.com.

The pooled prevalence of poor sleep quality in patients with cancer was 57.4% [95% confidence interval (CI): 53.3–61.6%], and higher prevalence rates were reported among patients with gynecological cancer (*p* < 0.01) (56), which results in the circadian disruption. At the molecular level, circadian regulation is mediated by core circadian genes and their protein products, molecular mechanisms and physiological importance of circadian rhythms. The molecular clock mechanism initiates with BMAL1 (Brain and Muscle ARNT-Like Protein 1) and CLOCK (Circadian Locomotor Output Cycles Kaput) forming heterodimeric complexes that bind E-box motifs within promoters of target genes, including clock-controlled genes (CCGs) and negative regulators period (PER) and Cryptochrome (CRY) (Figure 1A). Accumulation of PER/CRY proteins ultimately suppresses CLOCK-BMAL1 transcriptional activity, establishing an autoregulatory loop fundamental to circadian rhythm generation (57). Nevertheless, the International Agency for Research on Cancer (IARC) in 2019 concluded that night-shift work was possibly carcinogenic (58). Epidemiologic studies have yielded conflicting results as to whether circadian clock disruption by night or shift work is carcinogenic (59, 60). Circadian rhythm disruption has been mentioned to be associated with breast cancer in women. No relevant studies have addressed gynecological cancer, either in cohort studies or basic studies, but this deserves further exploration.

#### 2.1.1 Clock gene dysregulation

Circadian oscillators coordinate the temporal regulation of cellular proliferation, DNA repair mechanisms, and redox homeostasis. Emerging evidence links circadian disruption to enhanced tumorigenesis and metastatic dissemination through dysregulation of cancer stem cell maintenance and niche remodeling (61). Notably, BMAL1/CLOCK dimerization and cyclical expression of PER1-3 genes regulate critical oncogenic pathways across multiple malignancies. Experimental models demonstrate BMAL1 depletion accelerates endometrial carcinogenesis through mTOR pathway activation. As a circadian rhythm synchronizer, melatonin has been confirmed to regulate central and peripheral clock genes by up-regulating or down-regulating specific clock genes to control cell cycle, survival, and repair mechanisms (62). Hypoxia exposure causes dysregulation of ovarian circadian clock protein (CLOCK, BMAL1, and E4BP4) expression, which mediates female reproductive dysfunction by impairing LHCGR-dependent signaling events (63). Melatonin exhibits significant antitumor effects by modulating various signaling pathways, promoting apoptosis, and suppressing metastasis in breast cancers and gynecological cancers, including ovarian, endometrial, and cervical cancers (64–66). Ovarian malignancies exhibit prognostic correlations with PER1-3 downregulation, suggesting circadian gene dysregulation may influence therapeutic responses (67). Clinical studies in rectal, pancreatic, and endometrial carcinomas, as well as lymphoblastic leukemia, reveal that chronomodulated chemotherapy regimens optimize drug efficacy while minimizing toxicity (68). Therefore, this circadian rhythm disorder caused by obstructive sleep apnea is believed to create a microenvironment conducive to the development of ovarian cancer by promoting genomic instability, impairing DNA damage repair, and allowing cells to proliferate uncontrollably—all of which are key characteristics of ovarian cancer development.

#### 2.1.2 DNA repair impairment

The tumor microenvironment (TME) exhibits circadian-modulated immune dynamics, where PER1 expression inversely correlates with infiltrating B lymphocytes, macrophages, and neutrophils in ovarian cancer (69). PER2 and BMAL1 deficiency in ovarian tumors impairs homologous recombination repair (HRR) capacity. PER2 deficiency, in particular, regulates key downstream genes involved in cell cycle control (cyclin A, cyclin B1, cyclin D1, cyclin E) and DNA damage response (p53, c-myc); the dysregulation of these genes disrupts genomic maintenance mediated by SIRT1, BRCA1, BRCA2, and TP53 (70–72).

### 2.2 Hypoxia-driven tumor plasticity

OSA is closely linked to gynecological cancers due to its hallmark pathophysiological feature—IH. Hypoxia plays a critical role in the progression of gynecologic oncology, such as cervical cancer, by activating adaptive cellular pathways that promote tumor survival, invasion, and resistance to therapy. Here are key mechanistic insights:

#### 2.2.1 HIF-1α/ERα synergy

Hypoxia (tissue pO_2_ < 10 mmHg), prevalent in 90% of solid tumors (73, 74), activates HIF-1-mediated transcriptional programs governing angiogenesis, glycolytic metabolism, and invasion (75). OSA-associated IH produces distinct HIF isoform regulation, with preferential stabilization of HIF-1α over HIF-2α (Figure 1B) (76, 77). Mechanistically, hypoxia impairs prolyl hydroxylase-mediated HIF-1α degradation, enabling nuclear translocation and heterodimerization with HIF-1β. Subsequent recruitment of CBP/p300 coactivators to hypoxia-response elements (HREs) drives the expression of >100 target genes critical for hypoxic adaptation (78–81).

Experimentally, IH demonstrates accelerated HIF-1α activation compared to sustained hypoxia (82, 83). In gynecologic malignancies, ERα stabilizes HIF-1α to potentiate epithelial-mesenchymal transition (EMT) (84). HIF-mediated VEGF induction promotes tumor neovascularization, though resultant vessels exhibit structural abnormalities that perpetuate hypoxia (85). The hypoxic TME fosters metabolic adaptation via Warburg-effect dominance, generating lactate-rich microenvironments that facilitate immune evasion (86). Concurrent HIF-1α-mediated upregulation of anti-apoptotic Bcl-2/Survivin and suppression of Bax expression enhances tumor cell survival (87). Hypoxia also induces TGF-β-driven EMT programs critical for metastatic dissemination (88), while promoting M2 macrophage polarization and PD-L1-mediated T cell exhaustion (89, 90). Clinical evidence indicates that intratumoral hypoxia is a significant predictor of poor disease-free survival in cervical cancer patients (91, 92). IH mimicking sleep apnoea increases spontaneous tumorigenesis and cancer progression in mice (93, 94). Therefore, there is a link between hypoxia and gynecological tumors, however, how sex-specific pathways, such as estrogen-mediated regulation of hypoxia-inducible factors (e.g., HIF-1α), might contribute to gynecologic tumorigenesis in OSA patients deserves to be further explored.

#### 2.2.2 Metabolic reprogramming

HIF-1α orchestrates metabolic switching through PDK1 upregulation, which phosphorylates and inactivates pyruvate dehydrogenase (PDH). This redirects pyruvate flux toward lactate production via HIF-1α: CEBP-β axis activation under IH conditions (95–97). Increased expression of ABCB1/ABCC1 transporter, along with downregulation of ABCA1, has been observed in tumors exposed to IH, correlating strongly with enhanced chemoresistance (98). Autophagy-related gene BECN1 exhibits a positive correlation with HIF1α expression, indicating potential synergistic adaptation mechanisms that support cell survival within hypoxic tumor microenvironments. In mice, IH-induced neuroinflammation and mitochondrial ROS damage can be ameliorated through activation of the PINK1-Parkin mitophagy pathway (99).

### 2.3 Microbiota-immune axis dysfunction

#### 2.3.1 Gut barrier breakdown

OSA-related hypoxia has been shown to induce intestinal hyperpermeability, as indicated by increased circulating levels of lipopolysaccharide (LPS) and LBP levels in pediatric cohorts (Figure 1C) (100). Gut dysbiosis in sleep-disordered breathing is characterized by an elevated Firmicutes/Bacteroidetes ratios and the predominance of endotoxin-producing genera (Klebsiella, Prevotella), which promote systemic inflammation through activation of the TLR4/NF-κB signaling pathway (101, 102). The female reproductive tract (FRT) microbiota interacts with the gut and with the urinary tract, defining a vagina–gut axis and a vagina–bladder axis, respectively (103, 104). The gut and vaginal microbiota secret metabolites like endotoxins, bile acids, lipopolysaccharides, genotoxins and conjugated estrogen, which can induce DNA damage and increase genomic instability—factors implicated in carcinogenesis and disease progression (105). Therefore, OSA-altered microbial metabolites (e.g., LPS) and genotoxins can promote tumorigenesis by inducing systemic inflammation (NF-κB activation), ROS-mediated DNA damage, and impaired epithelial barrier function—all established drivers in endometrial and ovarian carcinogenesis. This dysregulation may form a self-sustaining loop: dysbiosis amplifies systemic inflammation and genotoxicity, driving tumor initiation/progression, while cancer-associated inflammation and therapy further disrupt microbial homeostasis. However, these viewpoints still require further research to clarify their potential mechanisms and clinical relevance.

#### 2.3.2 Tumor-associated macrophage polarization

The exposure of IH facilitates remodeling of TEM by inducing M2 macrophage polarization of adipose tissue macrophages (ATMs) and tumor-associated macrophages (TAMs), promoting regulatory T cell (Treg) expansion, and facilitating the adipocyte stem cell (ASC) accumulation (106). Intermittent hypoxia activates the NF-κB/HIF-1α pathway, increasing pro-inflammatory mediators like COX-2, CCL2, CXCL1, PGE2, and CSF1, these mediators recruit monocytes and neutrophils to the tumor microenvironment (TME), differentiating into TAMs and Tumor-Associated Neutrophils (TAN) (107–110). TAM and TAN can both lead to increased secretion of VEGF-A, thereby promoting cancer development (107). Sleep fragmentation-induced IL-10 secretion further reinforces an immunosuppressive TME, potentially facilitating tumor progression by inhibiting effective anti-tumor immune responses. In an animal model of IH, M2-like TAMs are activated in cervical cancer, contributing to a tumor-promoting microenvironment. IH caused by OSA can also cause the polarization of tumor-associated macrophages in mice (111). OSA appears to be closely linked to tumor initiation and progression; however, the mechanisms underlying its comorbidity with gynecological tumors—particularly the role of tumor-associated macrophages (TAMs)—remain underexplored.

## 3 Cancer therapy-exacerbated sleep disorders

### 3.1 Chemotherapy neurotoxicity

Chemotherapy-induced oxidative stress and autonomic dysfunction may serve as a critical bridge linking cancer treatment to the worsening of sleep disturbances, particularly OSA exacerbation. Chemotherapy-induced peripheral neurotoxicity (CIPN) constitutes a major dose-limiting complication in oncology practice, characterized by structural and functional damage to peripheral nerves (Figure 1D) (112).

#### 3.1.1 Paclitaxel-induced phrenic neuropathy

Chemotherapeutic agents directly exacerbate sleep-disordered breathing through neuro-autonomic pathways. Central to this phenomenon is paclitaxel-induced diaphragmatic dysfunction, occurring in 4–8% of gynecologic oncology patients due to microtubule stabilization disrupting phrenic nerve conduction (113–115). Approximately 60–70% of patients develop peripheral neuropathy following paclitaxel therapy, with severe manifestations (≥ grade 2) affecting 36% of elderly ovarian cancer patients and 20% of younger cohorts (112, 116).

#### 3.1.2 Chemotherapeutic resistance and oxidative stress crosstalk

Emerging evidence suggests a bidirectional relationship between chemotherapy-related oxidative stress and comorbid OSA, which may reciprocally exacerbate clinical outcomes. The emergence of multidrug resistance (MDR) during chemotherapy remains a major obstacle in cancer therapeutics, significantly compromising treatment efficacy, patient survival, and quality of life. This resistance phenomenon also impacts the clinical utility of reactive oxygen species (ROS) modulators, whether administered as monotherapy or chemo-sensitizing adjuvants (117). Notably, chemotherapeutic agents like paclitaxel and cisplatin—cornerstones of first-line therapy for ovarian carcinomas and other malignancies (118–120)—paradoxically induce therapeutic complications through ROS generation, causing collateral damage to normal tissues while modulating tumor redox dynamics. Doxorubicin exemplifies this pleiotropic interplay, exerting anticancer effects via DNA damage induction alongside complex cell death modulation through apoptosis, senescence, autophagy, ferroptosis, and pyroptosis pathways (121).

Emerging evidence suggests a bidirectional relationship between chemotherapy-related oxidative stress and comorbid OSA, which may reciprocally exacerbate clinical outcomes. Chemotherapeutic activation of hypoxia-inducible factor (HIF) signaling can augment carotid body chemosensitivity, a peripheral oxygen-sensing system critical for ventilatory and sympathetic regulation. Pathological hypersensitization of this system in OSA patients drives sympathetic overactivation and metabolic dysfunction, manifesting as refractory hypertension and insulin resistance (122). These autonomic perturbations are further aggravated by ROS-mediated chemoreflex hyperactivity and baroreflex suppression, creating a vicious cycle of hypertension induction (122–124).

Intriguingly, tumors may co-opt oxygen-chemo-sensing mechanisms analogous to carotid body physiology to facilitate survival and growth within hypoxic tumor microenvironments. This adaptation is mediated by HIF-2α—a transcription factor essential for carotid body development and hypoxic chemotransduction (125)—which also promotes chemoresistance in cancers such as high-grade serous ovarian carcinoma (HGSOC). HIF-2α exerts its protective role through TGFBI-dependent PI3K/Akt pathway activation, which suppresses apoptosis and enhances DNA repair, while its stabilization via the USP9X-HIF-2α proteostatic axis sustains cancer stem cell populations to drive tumor recurrence (126, 127).

Clinically, the IH characteristic of OSA creates a feedforward loop that amplifies both chemoresistance and autonomic dysfunction. IH disrupts HIF homeostasis by stabilizing HIF-1α while destabilizing HIF-2α, skewing redox balance toward ROS overproduction via pro-oxidant enzyme induction and antioxidant suppression (125). The resultant oxidative stress not only accelerates tumor adaptation to chemotherapy but also heightens carotid body chemosensitivity. This dual effect may synergize with chemotherapy-induced HIF activation to exacerbate hypertension and sympathetic overdrive during cancer treatment. These pathophysiological intersections underscore the need for combinatorial therapeutic strategies targeting HIF-ROS signaling networks to simultaneously mitigate chemoresistance and OSA-associated complications.

Collectively, IH-induced HIF-1α stabilization, ROS accumulation, and β-adrenergic signaling foster DNA repair upregulation, drug efflux amplification, and tumor cell survival—directly compromising chemotherapy efficacy (249).

### 3.2 Hormone deprivation effects on sleep

#### 3.2.1 Estrogen blockade and withdrawal

Evidence suggests that menopausal hormone therapy (MHT) may elevate the risk of gynecological malignancies such as endometrial cancer and ovarian cancer (128, 129). Systemic estrogen suppression via aromatase inhibitors (AIs) in ovarian and endometrial cancers accelerates sarcopenia progression, with 38.8% overall prevalence peaking in endometrial (43.6%) and ovarian (42.5%) malignancies (130–132). This muscle-wasting condition independently predicts poor treatment responsiveness—a critical determinant of survival in patients receiving hormone therapy or chemotherapy for advanced/recurrent disease (133). Interestingly, estrogen depletion may exacerbate sarcopenia progression, given estrogen’s pleiotropic roles in muscle homeostasis: preserving satellite cell function, enhancing membrane stability, and mitigating oxidative stress during mitochondrial dysfunction (134). This pro-sarcopenic effect is clinically consequential, as patients with dual diagnoses of gynecologic malignancies and sarcopenia exhibit significantly worse overall survival compared to those without muscle loss (135–138).

Notably, estrogen-deficient states—whether iatrogenic (AI-induced) or physiological (postmenopausal)—amplify vulnerability to upper airway myopathy under intermittent hypoxic stress, a hallmark of OSA. Ovariectomized models demonstrate sex-specific respiratory muscle deterioration under chronic intermittent hypoxia (CIH), partially reversed by estrogen replacement (139). These findings align with clinical observations of estrogen’s protective role in maintaining upper airway muscle tonicity and ventilatory drive (140), mediated through estrogen receptor α (ERα)-dependent regulation of mitochondrial bioenergetics (141). Consequently, AI-mediated estrogen suppression may inadvertently promote OSA by dual mechanisms: diminishing upper airway dilator muscle function (as evidenced by reduced genioglossus EMG activity post-therapy) (140) and exacerbating sarcopenia-related respiratory weakness. Therefore, when both gynecological cancer and OSA are present, the use of estrogen inhibitors may require careful consideration from multiple aspects.

#### 3.2.2 Fatigue, circadian dysregulation, and sleep architecture disruption

Cancer-related fatigue (CRF) in ovarian cancer manifests as a persistent, activity-limiting burden distinct from normal fatigue, intricately linked to sleep disturbances and neuroendocrine dysregulation. Longitudinal studies reveal sustained sleep impairment in ≥60% of epithelial ovarian cancer survivors (EOCS) at one-year follow-up, independent of depression yet correlated with diminished quality of life (142). General analyses based on weighting according to sample size showed a significantly positive correlation between fatigue and circulating levels of inflammatory markers, including significantly positive correlations between fatigue and elevated interleukin-6 (IL-6) (143). IL-6 levels consistently predict both poor sleep quality and fatigue severity, while aberrant cortisol rhythms—characterized by flattened diurnal variability and nocturnal hypercortisolemia—are mechanistically implicated in CRF pathogenesis (144–146).

Pelvic irradiation induces irreversible sleep architecture damage in gynecologic oncology patients, with 88% of irradiated cohorts developing OSA versus 67% in non-irradiated controls (147, 148). This increased risk of radiation-associated OSA likely results from fibrotic damage to upper airway tissues, compounded by preexisting sarcopenia. Crucially, pharmacological sleep aids have limited efficacy in improving long-term sleep quality in epithelial ovarian cancer survivors (EOCS), highlighting the importance of developing and implementing non-pharmacological management strategies (142). These intersecting pathways—cytokine-mediated inflammation, HPA axis dysfunction, and treatment-related anatomical changes—establish a self-perpetuating cycle wherein sleep disruption exacerbates fatigue, subsequently hindering physiological recovery and overall well-being.

## 4 Clinical challenges and biomarker detection

### 4.1 Diagnostic dilemmas

#### 4.1.1 Gender-specific OSA phenotypes: female-predominant hypopnea-dominant vs. male-predominant apnea-dominant OSA

Identifying sex- and gender-related differences in sleep research holds the potential to advance personalized care by improving diagnosis, treatment, and prevention of sleep disorders and related comorbidities. Besides, hormonal changes post-puberty has been proposed as a key factor contributing to adult sleep disparities (149, 150). Female sex hormones, such as estradiol and progesterone, are associated with increased sleep fragmentation, including frequent awakenings and prolonged wakefulness (150). The landmark Wisconsin Sleep Cohort Study reported OSA prevalence (AHI ≥5) in 24% of men and 9% of women (151). By 2013, meta-analyses revealed a mean OSA prevalence of 22% (range: 9–37%) in men and 17% (range: 4–50%) in women (151). Research indicates gender-specific phenotypes in OSA, with women more commonly exhibiting hypopnea-dominant OSA, while men tend to present with apnea-dominant forms (152). Potential reasons for these gender differences include anatomical and physiological variations: women have structurally more stable upper airways, men exhibit greater chemoreceptor sensitivity to hypoxic/hypercapnic stimuli, men display higher abdominal and neck fat deposition linked to OSA risk, and women more frequently experience partial upper airway obstruction (152). Hormonal influences may also play a critical role, as postmenopausal women show higher OSA prevalence than premenopausal women (151).

Clinically, male-to-female OSA referral ratios exceed those observed in population studies, suggesting underdiagnosis in women. Contributing factors include social stigma around snoring symptoms leading to fewer women seeking care (153, 154), though women diagnosed with OSA exhibit better treatment adherence. Gender differences in symptom presentation also exist at comparable AHI levels, women report less daytime sleepiness, habitual snoring, and witnessed apneas compared to men (153, 154). Polysomnographic differences further reveal that women exhibit similar OSA severity to men during REM sleep but milder events during NREM sleep (153), resulting in a higher proportion of REM-related respiratory events. Women also demonstrate more respiratory effort-related arousals (RERAs) and upper airway resistance syndrome (UARS) (155), which may be underrepresented by current scoring criteria, increasing the risk of underdiagnosis (156).

Collectively, anatomical, hormonal, behavioral, and diagnostic disparities likely amplify gender-specific OSA prevalence and clinical manifestations, necessitating further research to refine gender-sensitive diagnostic and therapeutic strategies.

#### 4.1.2 Fatigue overlap: distinguishing cancer-related fatigue from OSA symptoms

The overlap of fatigue-related symptoms presents a diagnostic challenge in CRF from OSA. CRF is persistent, intense, longer in duration, and not alleviated by rest as compared to more traditional fatigue (157, 158). Tumor- and/or treatment-associated cytokines have a proposed role in cancer-related fatigue via effects on central nervous system pathways that elicit vegetative behaviors (159–162). Supporting such hypotheses are findings that fatigued breast cancer survivors demonstrated significantly higher elevations of cytokines including IL-1ra, TNF-α, sTNF-RII, IL-6, and neopterin than non-fatigued survivors, and circulating levels of IL-6, IL-1ra, and neopterin have been associated with fatigue in a quantitative review of cancer patients (159–162). Given the strong relationship between excessive daytime sleepiness and complaints of fatigue, depression, and/or insomnia, these symptoms are also more commonly reported by women. Complaints of fatigue, tiredness, or lack of energy may be as important as that of sleepiness to OSAS patients, among whom women appear to have all such complaints more frequently than men (163). The diagnosis of OSA should not be excluded based only on a person’s tendency to emphasize fatigue, tiredness, or lack of energy more than sleepiness (163). Research has shown that 93% of patients with head and neck tumors experience daytime fatigue. Among these patients, 79% had undergone radiotherapy prior to the sleep study, and of this subgroup, 88% were diagnosed with OSA. In contrast, among patients who had not received prior radiotherapy, 67% were found to have OSA (148). These findings suggest a potential link between tumor treatment-radiotherapy and increased OSA prevalence, underscoring the need for routine sleep assessments in this patient population.

### 4.2 Prognostic markers

#### 4.2.1 Circulating exosomal miRNAs

Exosomal miR-210 is an important regulator of cell function via its downregulation of mitochondrial biogenesis, which attenuates oxidative phosphorylation demand (164, 165). The serum concentration of miR-210 was higher in individuals with OSA, and studies have demonstrated a potentially major role of miR-210 in mediating OSA-induced vascular risk (166). The mechanisms underlying OSA risk may be modulated by SREBP2—miR-210—induced mitochondrial dysfunction in endothelial cells (ECs), providing compelling evidence that miR-210 may be a suitable candidate as an OSA biomarker and a therapeutic target for interventional studies (166). miR-210 expression is upregulated in response to hypoxia in epithelial ovarian cancer specimens and cell lines, with an association to HIF-1α overexpression (167). Furthermore, upregulated miR-210 promoted tumor growth *in vitro* via targeting PTPN1 and inhibiting apoptosis (167). Sevoflurane and desflurane both promoted SKOV3 cancer cells (an ovarian cancer type) and malignancy via miR-210 (167), highlighting its potential role as a hypoxia-associated predictor of platinum resistance. Therefore, perhaps miR-210 may act as a diagnostic marker for gynecological cancer comorbid with OSA.

#### 4.2.2 Sleep EEG signatures

Slow-wave sleep (SWS) loss correlates with elevated IL-6 and shorter progression-free survival in cancer patients (168). Postoperative sleep disorders (PSD) are characterized by post-surgical alterations in sleep quality (169). Patients who undergo major surgeries experience lower sleep efficiency, disrupted sleep, decreased rapid eye movement (REM) sleep, and, in some cases, absence of the N3 sleep stage (170). OSA patients may have decreased SWS (171). There are also therapeutic methods targeting slow-wave sleep in female patients. SWS and REM sleep can be stimulated by prolactin (PRL) (172), which shares cellular effects exerted through the JAK/STAT signaling cascade, including survival, cell cycle progression, proliferation, migration, high metabolic rates, angiogenesis, and anti-apoptosis (173–176). Notably, renal sympathetic denervation has been shown to ameliorate inflammatory responses in a chronic obstructive sleep apnea (OSA) model via JAK/STAT pathway modulation, demonstrating conserved mechanistic cross-talk between neurohumoral regulators and inflammatory pathophysiology in sleep disorders (177). IL-6 has been shown to enhance non-rapid eye movement (NonREM) sleep in rats and slow wave activity during SWS in humans (166, 178, 179). S-ketamine can improve the prognosis of patients undergoing gynecological laparotomy by improving SWS (180).

The co-occurrence of slow-wave sleep (SWS) deficits and IL-6 elevation manifests as a shared phenotypic feature in both populations with obstructive sleep apnea (OSA) and post-gynecologic oncology patients, suggesting the potential utility of SWS/IL-6 profiling as discriminative biomarkers for OSA screening within gynecologic cancer cohorts.

## 5 Emerging therapeutic strategies

### 5.1 Microenvironment-targeted therapies

#### 5.1.1 Hypoxia-targeted interventions and nanoparticles

**Figure 2:**
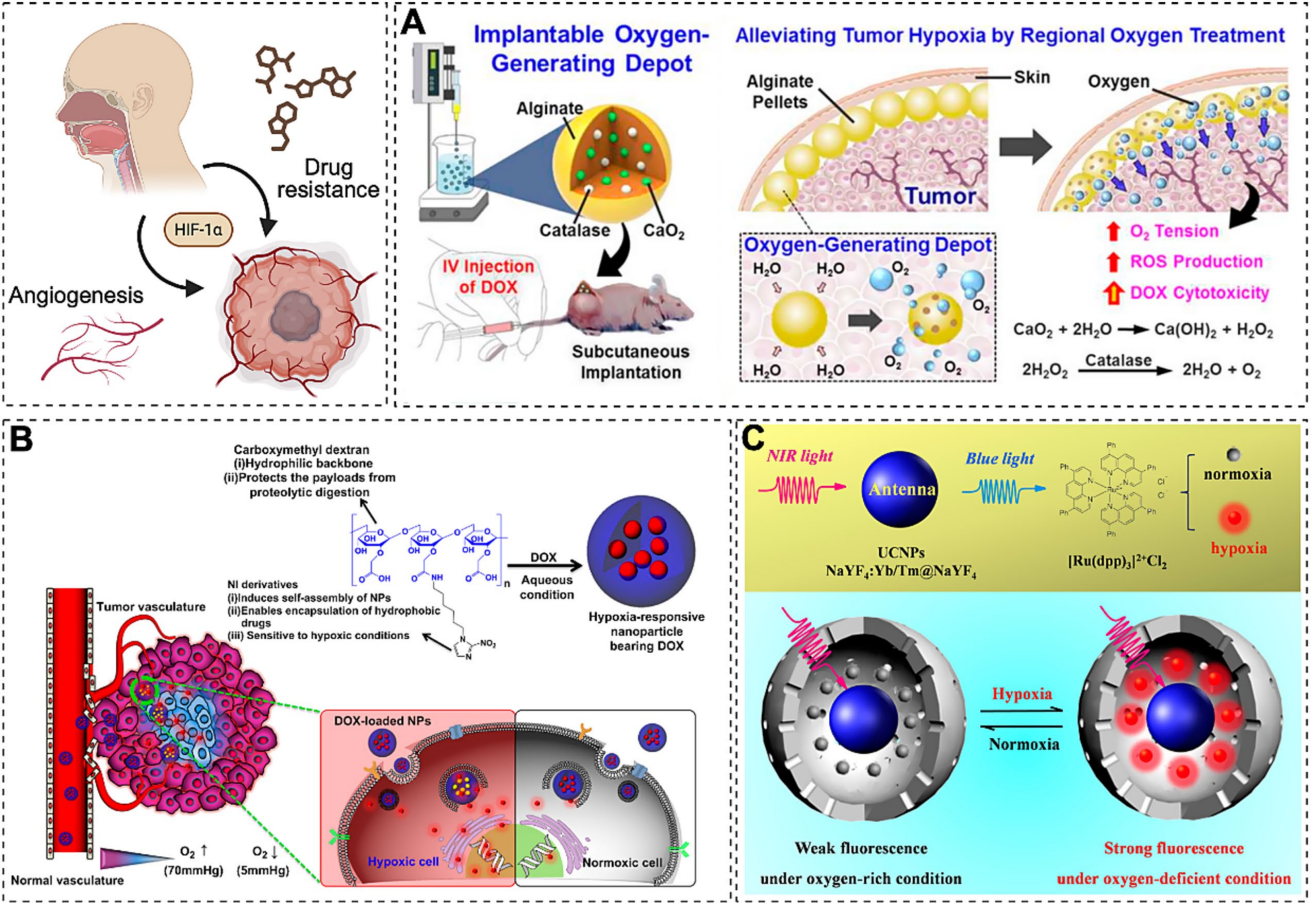
Emerging therapies target HIF-1α-driven VEGF and glycolytic enzyme upregulation to combat drug resistance. **(A)** Oxygen-delivering alginate pellet structure and DOX cytotoxicity enhancement in tumors (Reprinted with permission (126), licensed under CC BY). **(B)** EPR-mediated hypoxia-responsive drug release in tumors (Reprinted with permission (189), Copyright © 2013 Elsevier Ltd.). **(C)** NIR-activated UCNP-Ru hybrids enable real-time *in vivo* hypoxia imaging via photon upconversion (Reprinted with permission) (191).

The hypoxic tumor microenvironment drives malignant progression through HIF-1α-mediated upregulation of vascular endothelial growth factor (VEGF) and glycolytic enzymes (181–183). Emerging evidence indicates that IH induced by OSA exacerbates tumor hypoxia, potentially accelerating oncogenesis and chemoresistance. To counter these challenges, nanoparticle-based delivery systems have been engineered as multifunctional platforms for precision targeting of therapeutic agents, including small interfering RNA (siRNA), hypoxia-responsive nanotherapeutics, and conventional chemotherapeutic drugs (184). While current clinically approved nanomedicines primarily utilize passive tumor targeting via liposomal or polymeric carriers leveraging the enhanced permeability and retention (EPR) effect (185). Hypoxia-activated prodrugs such as mitomycin C remain limited by suboptimal clinical efficacy (186), necessitating innovative strategies for tumor reoxygenation and hypoxia modulation. Recent advancements in hypoxia-targeted nanotherapeutics focus on three synergistic approaches: tumor oxygenation via oxygen-generating biomaterials (e.g., alginate depots encapsulating catalase and calcium peroxide) (187, 188), hypoxia-responsive nanocarriers for spatiotemporally controlled drug release (e.g., doxorubicin-loaded hypoxia-responsive nanoparticles) (Figure 2A), and HIF-pathway inhibition using nanotechnology-enabled gene silencing (Figure 2B) (189). And the exploration of nanosensors which are capable of accurate diagnosis of hypoxic level is in urgent demand to estimate the malignant degree of cancer for subsequent effective and personalized cancer treatments (190). Concurrently, the development of hypoxia nanosensors has enabled dynamic monitoring of tumor microenvironment dynamics. For instance, Liu et al. (191) designed near-infrared (NIR)-activated upconversion nanoparticles (UCNPs) coupled with ruthenium-based complexes, achieving real-time *in vivo* hypoxia imaging through light conversion mechanisms (Figure 2C). Integration of nanotechnology with multimodal therapies demonstrates enhanced efficacy against resistant malignancies. Notable examples include reactive oxygen species (ROS)-responsive nanoplatforms co-delivering apatinib and doxorubicin for chemo-photodynamic synergy (192), alginate-optimized niosomes co-encapsulating doxorubicin and cisplatin to overcome ovarian cancer resistance (193) and albumin nanoparticles combining HIF-1α siRNA with methylene blue-mediated photodynamic ablation (194, 195). Parallel developments in OSA management reveal therapeutic interactions, where short-term continuous positive airway pressure (CPAP) withdrawal may enhance radiosensitivity through IH preconditioning, while prolonged CPAP application suppresses oncogenic pathways (e.g., JUN/MYC/SMAD3) in specific patient subsets (196). Notably, nanotechnology now enables both therapeutic intervention and physiological monitoring in OSA-related comorbidities (197). Key advances include CNS-targeted hesperidin delivery for leptin sensitization in obesity-associated sleep-disordered breathing, and ROS-responsive nanotherapeutics attenuating intermittent hypoxia-induced cognitive impairment through NRF2/KEAP1/HO-1 pathway modulation (198, 199). hypoxic tumor (200–202). This disparity underscores the necessity for personalized nanomedicine strategies that simultaneously address tumor hypoxia heterogeneity (quantified via nanosensors) and patient-specific physiological barriers.

#### 5.1.2 Fecal microbiota transplantation

The impact of gut microbiota on the body’s immune system and hormonal balance is significant, dysbiosis of gut microbiota has been associated with the promotion of common gynecological diseases such as PCOS, endometriosis, and malignant tumors (203). Recent studies have shown that sleep insufficiency/deprivation alters the composition of gut microbiota in both humans and rodents (204, 205). Fecal microbiota transplantation (FMT) is being evaluated for its potential to enhance immune checkpoint blockade therapy in clinical studies (primarily for metastatic melanoma) (206). HPV is linked to cervical cancer (207). In a study by Xu et al. (208), ovarian cancer cells transplanted into mice with gut microbiota dysbiosis exhibited increased xenograft tumor sizes; this dysbiosis stimulated macrophages, elevating circulating interleukin (IL)-6 and tumor necrosis factor-α levels, thereby inducing ovarian cancer epithelial-mesenchymal transition. *Lactobacillus plantarum* HL2 mitigated PCOS-like pathological changes in ovaries (209). Melatonin ameliorates cadmium-induced intestinal mucosal damage by scavenging ROS and increasing goblet cell numbers, while *Akkermansia muciniphila* regulates melatonin production via increased enterochromaffin cells (210). Melatonin acts as a safe nutraceutical to limit skeletal muscle frailty, prolong physical performance, and target mitochondrial function by reducing oxidative damage (211–213). At week 12, FMT recipients showed higher insomnia remission rates versus controls (37.9% vs. 10.0%; *p* = 0.018), with significant reductions in ISI, PSQI, GAD-7, ESS scores, and cortisol (*p* < 0.05), while controls displayed no changes (214). Targeting microbiota may alleviate sleep deprivation effects (215). OSA alters gut microbiome diversity, and IH-induced GM changes can mediate sleep disturbances independently (215). Melatonin supplementation in NLRP3 KO mice delayed sarcopenia onset in aged animals, suggesting gut microbiota regulation (including FMT) as a potential sleep disorder therapy. The balance of gut microbiota benefits both gynecological tumors and OSA (210). Thus, exploring mechanistic connections and therapeutic synergies between these conditions is warranted.

### 5.2 Circadian optimization

Many cellular functions including the cell cycle and cell division are, at least in part, controlled by the molecular clock components (CLOCK, BMAL1, CRYs, PERs), it has also been expected that appropriate timing of chemotherapy may increase the efficacy of chemotherapeutic drugs and ameliorate their side effect (216, 217). Chronomodulated chemotherapy, such as timing oxaliplatin to PER3 expression peaks, improves endometrial cancer response and reduces treatment toxicity (218). OSA-related circadian rhythm disorders are linked to hypoxia-induced HIF1α augmentation, with HIF1α mRNA levels positively correlating with Bmal1, Cry1, and CK1δ expression (219). The combined expression of CRY1 and PER3 enhances prediction of severe OSA (220). In ovarian cancer, time-regulated 5-fluorouracil/leucovorin combined with radiotherapy is well-tolerated (221). Light therapy using blue wavelengths resets circadian phase, reducing cancer-related insomnia (222). While artificial light at night (ALAN) increases cancer risk and metabolic/mood disorders (222), blue light exposure disrupts cervical cell metabolism and reduces melatonin secretion via “darkness deficiency” (223, 224). Appropriately timed bright light exposure synchronizes biological rhythms and improves sleep–wake cycles, offering a nonpharmacological option for cancer patients (225).

### 5.3 Probiotic cocktails

Growing evidence has revealed the intimate relationship between the gut microbiota and anticancer treatments, including chemotherapy (226), radiotherapy (227), targeted therapy (228), and immunotherapy (229). Probiotics help maintain the integrity of the intestinal barrier and reduce chemotherapy drug-induced damage to the intestinal tract, thereby lowering the incidence of gastrointestinal side effects (230). Probiotics can improve patients’ quality of life by regulating the intestinal microbiota and reducing toxic reactions caused by chemotherapy drugs such as cisplatin. The early cytotoxicity of cisplatin depends on reactive oxygen species (ROS) generation, and probiotics enhance cisplatin efficacy by modulating the oxidative stress response (231). When probiotics (e.g., *Lactobacillus*) are combined with cisplatin, they induce tumor cell apoptosis, enhancing cisplatin’s anticancer effect (232). OSA-associated IH alters the gut microbiome, contributing to cardiovascular dysfunction; probiotics and prebiotics mitigate these effects by increasing cecal acetate concentrations (232, 233). Interestingly, the benefits of gut microbiota seem to manifest simultaneously in both tumor occurrence and development, as well as in the severity and complications of OSA.

## 6 Future directions

### 6.1 Mechanistic insights

#### 6.1.1 Single-cell multiomics

In cancer research, hypoxic tumor niches represent a critical focus. These oxygen-deprived regions are linked to tumor aggressiveness, therapy resistance, and immune evasion. Single-cell multiomics enables precise mapping of these niches and their interactions with neighboring cells. Sleep disorders impair immune function, potentially altering immune cell states within the TME. For instance, sleep deprivation may reduce T cell and natural killer (NK) cell efficacy, compromising immune surveillance and tumor clearance.

**Figure 3:**
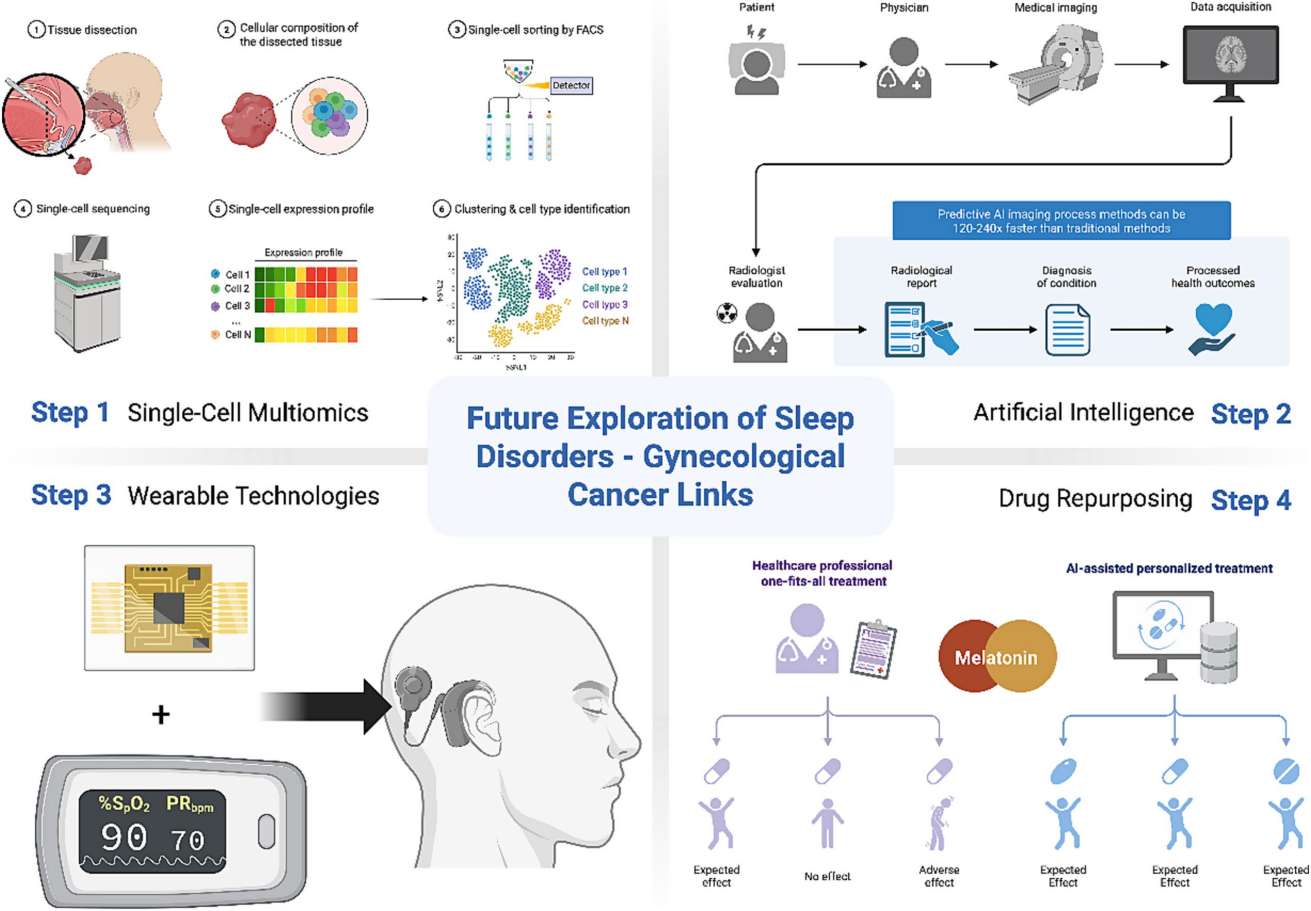
Future exploration of sleep disorders-gynecological cancer links. Step 1: Single-cell multiomics unravels dynamic tumor ecosystem complexities. Step 2: AI assists PSG scoring. Step 3: Wearable technologies monitor real-time SpO_2_ and activity data. Step 4: Repurposing melatonin agonists may offer safer circadian restoration and chemo-sensitization. Created with Biorender.com.

Single-cell multiomics simultaneously analyzes multiple molecular profiles (genome, transcriptome, epigenome, proteome) at the single-cell level, offering unprecedented resolution for studying complex systems like TME (Figure 3) (234). Utilizing this approach, Yao et al. (235) identified a tumor immune barrier (TIB) composed of SPP1+ macrophages and cancer-associated fibroblasts (CAFs) near tumor boundaries, influencing immune checkpoint blockade efficacy. Hypoxia-driven SPP1 expression promotes macrophage-CAF interactions, stimulating extracellular matrix remodeling and TIB formation, limiting immune infiltration in tumor cores. This technology elucidates hypoxic niche–sleep-disrupted immune cell interplay, identifying therapeutic targets to enhance immunotherapy.

#### 6.1.2 Artificial intelligence

Artificial intelligence (AI) enables “big data” analysis combining clinical, environmental, and laboratory metrics to advance sleep disorder understanding. Its medical applications include clinician-assisted image interpretation, health system workflow optimization, and patient self-monitoring (236). In sleep centers, AI assists polysomnography (PSG) scoring (Figure 3) (237). Tumor genomics identifies mutations and expression patterns for personalized treatment, while AI predicts therapeutic responses and drug targets. Esophageal pressure (Pes) monitoring, the gold standard for assessing respiratory effort during apneas/hypopneas, is invasive and rarely used. AI models trained on PSG-derived features simulated Pes in 1,119 individuals (237), highlighting AI’s potential to integrate PSG, cytokine, and genomic data for multimodal predictive modeling.

### 6.2 Translational opportunities

#### 6.2.1 Wearable technologies

Smartwatch SpO_2_ monitoring via photoplethysmography is critical for tracking respiratory health in cancer patients, particularly those with chemotherapy/radiotherapy-related respiratory dysfunction or OSA (237). Circadian rest-activity metrics show moderate-to-strong associations with cancer patient overall survival (238). Leveraging wearable devices (e.g., Apple Watch, BioStamp, Dreem, Oura) and machine learning techniques, perioperative cancer monitoring and mortality risk prediction in advanced cancer patients can be achieved through continuous measurement of core physiological indicators including body temperature, average heart rate, step count, and blood oxygen saturation (239–241). Meanwhile, real-time SpO₂ and activity data concurrently facilitate early detection of respiratory compromise and sleep disturbances, enhancing therapeutic optimization (Figure 3) (242). Future advancements may integrate biomarkers such as heart rate variability and dermal thermoregulation patterns for holistic assessment. Ultimately, wearable data streams are projected to catalyze comprehensive diagnostic and therapeutic paradigm shifts for sleep disorders and gynecological malignancies.

#### 6.2.2 Drug repurposing

Traditional insomnia treatments (benzodiazepines/nonbenzodiazepines) carry risks of cognitive impairment, falls, and dependence (243). Safe alternatives include slow-release melatonin (Circadian) and synthetic agonists (ramelteon, tasimelteon, agomelatine) (Figure 3) (244). Agomelatine, a MT_1_/MT_2_ agonist and 5-HT_2C_ antagonist, improves depression-related sleep without cognitive side effects (245). Melatonin enhances tamoxifen efficacy by up-regulating estrogen receptors and activating PKC/PKA pathways via MT_1_-mediated p27^Kip1^ induction (246–248). Repurposing melatonin agonists may offer safer circadian restoration and chemo-sensitization, though clinical validation remains essential.

## 7 Conclusion

These intertwined mechanisms highlight sleep as a modifiable therapeutic target in gynecologic cancer. Integrative strategies—like hypoxia-targeted nanomedicine (e.g., hypoxia-targeted nanoparticles), chronotherapy (e.g., circadian-optimized immunotherapy timing), and microbiota modulation (probiotics)—offer promising paths to improve outcomes through personalized, interdisciplinary care.
